# Diseases of the Nose

**Published:** 1887-12

**Authors:** W. S. Kendrick

**Affiliations:** Atlanta, Ga.


THE ATLANTA MEDICAL AND SURGICAL JOURNAL.

VOL. IV. DECEMBER, 1887. NO. 10.

### Original Communications.

### DISEASES OF THE NOSE.

BY W. S. KENDRICK, M. D., ATLANTA, GA.

It is the intention of this paper to speak mainly of hypertrophies of the turbinated bones and deviations of the septum, which seriously interfere with the physiological functions of the nasal cavities, and are accompanied by such pathological changes as require surgical means for their removal.

Probably in no department of medicine and surgery for the past few years have greater changes taken place than in the etiology and treatment of diseases of the nose and throat.

Only a few years ago the leading specialists, both in this country and Europe, in diseases of these cavities, confined themselves mainly to inflammations of the pharynx and larynx, usually the latter, thereby ignoring in a large measure diseases of the nose proper.

It has only been within the last two or three years that advanced investigators have turned their attention from the throat, claiming, as a result of their research and experience, the nose as being the seat and cause of most of the diseases connected with the throat; that the nose should be treated primarily and the pharynx and larynx secondarily—the cause being in the nose and the effects in the throat. Whether this be wholly true or not, there is no doubt that proper treatment, after a correct diagnosis and a true pathological knowledge of the disease as it exists, will enable us to relieve many cases of nasal catarrh and kindred affections that have heretofore been considered incurable.

Chronic nasal catarrh, in some form, whether light or grave, exists in a very large percentage of all persons arrived at the age of maturity. The reasons of this are quite obvious, as the nasal mucous membrane, from its situation, its great exposure to the atmospheric changes of heat and cold, and to the irritating influences of dust, smoke, etc., is the most liable of all the mucous membranes of the body to take on catarrhal inflammations.

It might be well to look briefly into the anatomy of the nasal cavities before attempting a description of the abnormalities of the parts under consideration. The cavity of the nose from the anterior to the posterior nares is divided by the septum, bony behind and cartillaginous in front. On the outer side of each nostril and opposite the septum are the three turbinated bones—superior above, inferior below, and the middle between them. Below the inferior, the largest of the three, and the floor of the nose is the inferior meatus, the main inspiratory tract, and through which principally instruments are passed and medicines applied to the surrounding parts. Between it and the middle turbinated bone and the septum is the middle meatus, and above that the superior or upper meatus.

The whole of the cavity of the nose and the accessory cavities is lined with mucous membrane, thicker and more vascular over some portions of the septum and turbinated bones than others.

Beneath the mucous membrane proper, especially on the middle and lower turbinated bones, and made up largely of venous sinuses, is what is known as the sub-mucous tissue, erectile in its character, the sinuses rapidly filling and emptying themselves from mere position or exposure, and entering largely as a factor in the various congestive diseases of the nose. The air as it passes through the nose to the lungs is moistened, warmed and purified by the arrest of particles of dust, or other extraneous substances, and whatever would obstruct for any length of time the free passage of air through the nose would interfere seriously with the proper functions of the part.

It is not the object of this paper to speak specially of acute inflammation of the nose, but the more chronic ones, that class of cases so often met with in every-day practice, known as "chronic catarrh," or "cold in the head," and which gives the physician so much trouble and the patient so much anxiety.

It is impossible, absolutely so, to give a correct opinion without a thorough knowledge of the anatomy of the parts in health, and also the pathological conditions so often found where chronic inflammations have existed for any length of time. Whether in anterior or posterior rhinoscopy, a good light is indispensable, as it would be folly to attempt an opinion without a thorough inspection of all the parts. Anterior rhinoscopy is easy, neither is it difficult to inspect thoroughly with reflected light the larynx, but posterior rhinoscopy is difficult, and can only be successfully acquired by much patience and constant application.

As said before, it is our intention to treat of the more chronic troubles of the nose and to dwell more upon hypertrophies than the simpler forms of chronic rhinitis. In the latter the mucous membrane from continual congestion is somewhat thickened, mostly in its outer layers, but in true hypertrophy there is not only thickening of the membrane proper, but induration from hyperplastic changes that have taken place, not only in the mucous membrane, but in the sub-mucous tissues, the whole being once, twice or several times thicker than in its normal condition. There are two main pathological conditions or divisions of diseases of the nose, viz., hypertrophic and atrophic diseases, the former much more frequent and more favorable as regards treatment than the latter. The parts usually found hypertrophied are the anterior and posterior ends of the inferior turbinated bones, the ends of the middle turbinated bones and the lower portion of the septum. On inspection anteriorly the ends are found reddened and swollen, the mucous membrane being much thicker than in the normal state, bulging out towards the septum, and in many instances lying in contact with that of the septum, and either partially or almost completely filling up the nasal cavity, acting as a constant, continual source of irritation to the mucous membranes of the pharynx, larynx and other accessory cavities, the irritation extending up the nasal duct, producing a thickening of its lining membrane and partial or complete stenosis, the same condition existing at the pharyngeal mouth of the eustachian tube, or extending along the canal into the middle ear, producing partial or complete deafness, tinnitus aurium and other distressing symptoms. The almost constant apposition of the hypertrophied mucous membranes of the turbinated bones and septum will in many cases produce the most distressing neuralgias of the head, face, ears, eyes, etc., increasing or diminishing as the congestive pressure in the nose is increased or lessened. Out of several cases treated in the last few months, the writer remembers one particularly, where neuralgia of the face and head had existed for a number of years, better at times and worse at others, accompanied by catarrhal symptoms, which increased as the years rolled by, until the pain became so frequent and acute as to render the patient miserable, when on examination there was found a great deviation of the septum in the left nostril with hypertrophy of the anterior end of the inferior turbinated bone; the deviation was removed with Bosworth's saw, the hypertrophy touched with the galvano-cautery, and almost immediately the neuralgia passed away and the catarrhal symptoms in a large measure subsided. There is no question in this case that the distressing symptoms were produced by mere pressure, and when that was taken off by surgical means, the patient was relieved.

The thickening over the turbinated bones and septum is not always uniform, but under the use of cocaine is sometimes found in patches, the result of more advanced hyperplasia in some portions than others. While hypertrophies are found mainly on the ends of the bones, still the entire free border, especially of the lower bone, is often found enlarged, projecting towards the septum and floor of the nose. One of the first evidences of hypertrophy of the tissue of the nose is difficulty of inspiration through the nasal cavities, the patient breathing largely through the mouth, thereby interfering with the general health and comfort. The air is carried to the bronchial tubes and lungs without being properly heated and moistened by the mucous membrane of the nose and relieved of floating particles of dust and other irritating substances. Besides the inflammation extending to the pharynx, larynx and bronchial tubes from mere continuity of surface, they are constantly irritated by the mere passage of air through them in all persons who habitually breathe through the mouth for reasons already given. The hypertrophied ends of the turbinated bones posteriorly do not always present the same appearance as the anterior; instead of the red, usually a swollen, grayish mass is found, almost filling up the posterior nares, or projecting backward into the pharynx. It is much more difficult to reach and treat that class of cases than the anterior variety on account of mere position.

There are two methods of determining almost positively whether a patient is suffering with simple chronic rhinitis or hypertrophic rhinitis, viz., by the probe and cocaine. If the former disease, and on inspection the anterior end of the turbinated bone is swollen, by firm pressure with the probe, the membrane can be pushed back against the bone, but when the probe is removed the recoil is slow and sluggish, for the reason there is a certain amount of thickening of the mucous membrane, especially of its outer layers, while in true hypertrophy firm pressure with the probe makes but little impression on the tissues, as not only the mucous membrane, but sub-mucous tissues have become permanently indurated and thickened from an increased growth of connective tissue, the result of a long-continued inflammation.

Hydrochlorate of cocaine applied to the membranes in chronic rhinitis causes the swelling to almost entirely disappear by its constringing effects on the blood-vessels, but when applied to true hypertrophies makes but little impression from the same reasons as given for the probe, viz., the connective tissue growth is not only around the vessels, but in the walls themselves, rendering them less contractile than before. Chronic nasal catarrh in some form, and due usually to hypertrophies of the turbinated bones, to deviations, or growths on the septum, is common in a large majority of the people of this country, growing possibly better in summer, but with each succeeding winter becoming worse.

The aggravating question has presented itself to most physicians, "What can be done?" If pharyngitis or laryngitis, shall these be treated primarily, or shall we look into the nose to find, in many cases, the true cause of the trouble, and, if found, remove it? If the ends of the turbinated bones are swollen to such an extent as to interfere with nasal respiration, or to lie when fully congested against the septum or floor of the nose, the method of treatment by insufflators, atomizers, and the direct application of astringents, etc., to the cavity of the nose will fail, in a large measure, in the hands of the general practitioner and the most accomplished specialist to give that relief which both the patient and physician so earnestly desire. They may be held in partial abeyance, but cannot be removed except by mechanical or surgical means. The usual method of treatment for the last year or so for hypertrophies over the turbinated bones is either the galvano-cautery, or some form of escharotic, preferably the former, if convenient, as the cautery points can be introduced cold, and applied directly to the diseased parts before the heating takes place and withdrawn in the same way. Of the escharotics chromic acid is the one most used on account of its being less liable to produce such destruction of tissue as nitric acid in the neighboring parts. No extensive applications of either chromic acid or the galvano-cautery should be made to the septum, as wounds in that part heal very slowly. If chromic acid be used, a thin, flattened probe, either heated and dipped in the acid crystals, or one side of the probe covered with hot sealing wax, and by being pressed against the acid enough will adhere to the wax to make a thorough and efficient application. Chromic acid, in the hands of an experienced operator, is an effective remedy to destroy hypertrophies of the turbinated bones, great care being taken to apply it only to the diseased surfaces. In the hands of an inexperienced operator, either it or the galvano-cautery would become a dangerous experiment. In no case should two opposing surfaces in close proximity be touched at the same time, as adhesion of the two raw surfaces might take place. In small hypertrophies or in chronic rhinitis, glacial acetic acid is often used, though it is much less effective than chromic, it taking seven or eight applications to produce the same destruction of tissue as the chromic or galvano-cautery. The deep eschar produced by acids or cautery usually comes away in a few days, the parts beneath healing almost by the time it is removed. It may be necessary to repeat the applications, which should not be done under two or three weeks, usually taking a much longer time to get the full effects of the treatment. The tendency of scars is to contraction, and it is to the destroyed tissue and the contractile powers of the scar made by the escharotic that the thickened mucous membrane on either side is drawn down and away from the septum.

In brief, whatever escharotic will destroy a sufficient amount of the redundant tissue and leave a scar whose contractile powers shall be able to draw away from the septum what redundancy may be left around the margins, so as to leave a free, open nostril, will accomplish the desired ends. The treatment for deviations and growths on the septum can be dispensed with in a few words.

The stronger acids and cautery should only be applied to the outer walls of the nasal cavity. There is scarcely an individual whose septum lies exactly in the median line. Growths, deviations, etc., should be removed with the knife, scissors, Bosworth's saw, or the surgical engine, the two latter being probably the best, great care being taken afterward to see that adhesions do not take place between the outer walls and septum. In posterior hypertrophies it may become necessary, in addition to the means mentioned, to use either the electric wire or cold snare, but the large majority of both anterior and posterior enlargements can be removed in the same way. The amount of pain and hemorrhage attending the various operations amounts to very little since the introduction of cocaine. Applied a few moments before the operation, no pain, only an unpleasant sensation, is felt and the procedure can be easily borne.

It is impossible, in a paper like this, to describe accurately the diseases under consideration, without proper drawings of the turbinated bones and septum, but in closing, the writer will not hesitate to say that in deviations the septum and chronic hypertrophies, both of which are very common, and the cause in many instances of naso-pharyngeal catarrh, surgical measures alone in the first instance, followed afterward by whatever treatment may be desired, promise our patients any relief from maladies so frequent and so annoying.

